# Excessive Extracellular Ammonium Production by a Free-Living Nitrogen-Fixing Soil *Clostridium* sp. Strain

**DOI:** 10.3390/microorganisms12122634

**Published:** 2024-12-19

**Authors:** Soyeon Park, Jeonghwan Jang

**Affiliations:** Division of Biotechnology and Advanced Institute of Environment and Bioscience, Jeonbuk National University, Iksan 54596, Jeonbuk, Republic of Korea

**Keywords:** free-living soil diazotroph, biological N fixation, soil *Clostridium* species

## Abstract

A Gram-positive, rod-shaped, and obligate anaerobic bacterial strain OS1-26 was isolated from apple orchard soil in Iksan, South Korea. Interestingly, strain OS1-26 was observed to possess the functional genes involved in biological nitrogen fixation (BNF), including *nifH*, which was actively transcribed during the anaerobic cultivation with excessive production of extracellular NH_4_^+^ despite of presence of other fixed N nutrients. The BNF of strain OS1-26 was distinguished from the other well-known *Clostridium* diazotrophs, such as *C. pasteurianum* and *C. acetobutylicum*. The altruistic N-fixing ability of the strain may play a pivotal role in providing N nutrients to the microbial community and plants in the soil ecosystem. The microorganism grew at 25–35 °C (optimum 30–35 °C) and pH 5.0–8.0 (optimum 6.0–8.0) but was not able to grow in the presence of >0.5% NaCl. The major cellular fatty acids of strain OS1-26 were C_16:0_, C_14:0_, and the summed feature consisted of C_16:1_ ω7c and C_16:1_ ω6c (35.63%, 25.29%, and 18.84%, respectively). The 16S rRNA phylogeny indicated that strain OS1-26 is a member of the genus *Clostridium*, and the closest species are *C. aciditolerans*, *C. nitrophenolicum,* and *C. thailandense,* with 16S rRNA sequence similarities such as 99.71%, 98.52%, and 98.45%, respectively. In spite of the high 16S rRNA sequence similarity, strain OS1-26 showed overall genomic relatedness, such as the average nucleotide identity (ANI), and phenotypical features distinctly different from *Clostridium aciditolerans*. Although the species taxonomy of strain OS1-26 is undetermined within the genus *Clostridium* based on overall genomic and phenotypic properties, further studies on the soil bacterial strain would enhance our understanding of its taxonomic identity, ecological roles for the terrestrial soil N cycle, and the potential to be developed as a biological N fertilizer.

## 1. Introduction

Most nitrogen (N) exists in the atmosphere as nitrogen molecules (N_2_). Thus, fixation of the gaseous nitrogen into a biologically available form, such as ammonium (NH_4_^+^), is an essential process providing N nutrients to ecosystems on the Earth [1]. Biological N fixation (BNF) is conducted by prokaryotes called diazotrophs, which are differentiated according to their lifestyle, such as symbiotic or free-living [2]. Although symbiotic diazotrophs, such as the ones forming legume nodules, have been extensively studied for enhanced agricultural production [3], free-living diazotrophs were relatively less studied because of a common perception that their contributions to N inputs are comparatively smaller and less important than those of symbiotic diazotrophs [2]. However, free-living N fixation would not be outweighed by symbiotic N fixation since the studies suggested evidence challenging the common perception. For example, many ecosystems do not carry many symbiotic N fixers, so free-living N fixers are likely a major source of N nutrients for ecosystems [4,5]. Moreover, many nodulated plant species do not always fix N_2_, and there is significant intraspecific variation in N fixation rates among them. Thus, the previous studies estimating symbiotic N inputs via the presence or absence of putative symbiotic N_2_-fixing plants would not be reliable [6,7,8]. The other studies estimated symbiotic and free-living N fixation rates at a large scale, such as at the biome level, and suggested that free-living fixation could contribute a greater proportion of N inputs than does symbiotic fixation [9].

The genus *Clostridium* includes diverse members of Gram-positive, rod-shaped, spore-forming, and obligate anaerobic bacteria in soils, water, host organisms, and other ecological habitats. The genus is composed of approximately 235 species and subspecies, and a few of them were reported to show beneficial effects on plants, such as phosphate solubilization and BNF [10]. *Clostridium pasteurianum* is the first-reported free-living N-fixing bacterium isolated by Winogradsky more than 100 years ago, and in addition, several other species of *Clostridium,* such as *C. acetobutylicum*, *C. acidisoli*, *C. akagii*, *C. beijerinckii*, *C. butyricum*, *C. formicoacelicum*, *C. hungatei*, and *C. kluyverii,* were also found to be N fixers [11,12]. The capability of N fixation in *Clostridium* seems to be not rare. Thus, there would be more novel N-fixing *Clostridum* species or an unnoticed N-fixing capability of the existing *Clostridium* species waiting to be discovered in future studies.

All the catalytic and regulatory enzymes involved in N fixation are encoded by the *nif* genes, and the nitrogenase complex is the primary enzyme encoded by the *nif* cluster, which is in charge of reducing N_2_ to NH_4_^+^ [13]. The *nifH* gene encoding the iron protein subunit in the nitrogenase complex is highly conserved among N-fixing bacteria and widely used as a marker gene to study the diversity and evolution of diazotrophs [14,15]. Given the expensive energy costs of N fixation by nitrogenase (16 ATP for the reduction of a mole of N), N fixation by diazotrophs is downregulated by the availability of external N sources, such as amino acids and NH_4_^+^, at the transcriptional or posttranslational level [16]. From a teleological point of view, it makes sense that diazotrophs use external N nutrients in favor of fixed N to save energy costs for BNF. Interestingly, a unicellular diazotroph in the ocean producing extracellular NH_4_^+^ via BNF despite the availability of fixed N nutrients has been reported previously [17], suggesting that the inefficient BNF of the diazotroph would provide benefits for its niche expansion and others in the microbial community. Soil diazotrophs that continue to excrete NH_4_^+^ even when fixed nitrogen nutrients are available would provide several unique benefits in agricultural and ecological contexts, such as a continuous N supply to plants and an enhancement of the soil microbiome and health.

Nitrogen (N) is a critical macronutrient that drives primary productivity in terrestrial ecosystems, playing a pivotal role in plant growth and soil health [18]. However, the ecological impact of N is complex, as its availability and forms can significantly influence microbial dynamics, nutrient cycling, and ecosystem functioning [19]. BNF, carried out by both free-living and symbiotic microorganisms, is a key process that replenishes bioavailable N in soils. Among these, novel free-living N-fixing bacteria are of particular interest due to their ability to enhance soil fertility without requiring external N inputs [16]. Such a diazotrophic strain, exhibiting the unique ability to continuously produce extracellular ammonium regardless of the presence of fixed N compounds in its environment, may be of great interest. This characteristic could disrupt typical feedback mechanisms that regulate microbial N fixation, leading to localized enrichment of bioavailable N. Consequently, this may alter microbial community structures and nutrient cycling processes within the soil ecosystem. While such a strain offers significant potential advantages for sustainable agriculture, it may also pose risks, such as unintended N leaching or shifts in microbial competition dynamics [20]. Further exploration of its ecological implications is essential for understanding its role in N management strategies and the resilience of soil ecosystems.

Here we report a free-living *Clostridium* sp. strain OS1-26 producing excessive extracellular NH_4_^+^ during anaerobic cultivation in a complex medium containing various fixed N nutrients. A whole-genome sequence analysis indicated that strain OS1-26 possessing the *nif* gene cluster is a member of the genus *Clostridium*. However, the species taxonomy of strain OS1-26 was not determined due to its overall genomic and phenotypic traits being distinctly different from the other previously recognized *Clostridium* species. This study provides the detailed genomic and phenotypic features of the soil bacterial strain OS1-26, including its unique BNF characteristic, which can be further studied for its roles in the terrestrial soil N cycle and the potential for being developed as a biological N fertilizer.

## 2. Materials and Methods

### 2.1. Bacterial Strains

Strain OS1-26 was obtained from surface soil (0–2 cm depth) collected at an apple orchard located in Iksan-si, Jeollabuk-do, Republic of Korea (35°58′44.03″ N 127°3′31.33″ E). The soil sample in the apple orchard contains 23 g of organic matter kg^−1^, 426 mg of available phosphorus kg^−1^, 1.15 cmol(+) K kg^−1^, 1.1 cmol(+) Ca kg^−1^, 0.9 cmol(+) Mg kg^−1^, and 0.14 electrical conductivity (dS/m), with a pH of 7.5 in the soil. The taxonomy of the apple orchard soil is clay loam. The contents of NO_3_^−^ and NH_4_^+^ were 32 mg N kg^−1^ and 56 mg N kg^−1,^ respectively, and the NO_2_^−^ was below the threshold for detection. For the isolation of strain OS1-26, 2 g of the soil sample were mixed with 10 mL of phosphate-buffered saline (PBS, pH 7.0), and 100 µL of the 1:100 diluted soil suspension was spread onto the surface of an R2A agar plate (MB cell, Seoul, Republic of Korea). After anaerobic incubation at 28 °C for 5 days using a GasPak EZ Anaerobe system (BD, Franklin Lakes, NJ, USA), the colonies that formed on the plate were re-streaked for purity onto new R2A agar plates to obtain well-isolated pure single colonies. The pure single colony of strain OS1-26 was selected for extracellular NH_4_^+^ accumulation to be observed via a colorimetric plate screening method described below.

Pure cultures of *Clostridium pasteurianum* KCTC 1674^T^ and *Clostridium acetobutylicum* KCTC 1790^T^ were purchased from the Korean Collection for Type Cultures (Jeongeup-si, Republic of Korea) to test their extracellular NH_4_^+^ production under the identical experimental conditions used for strain OS1-26.

### 2.2. Screening of Strains Producing Excessive Extracellular NH_4_^+^

To test extracellular NH_4_^+^ production of the soil isolates, each of the obtained pure single colonies was inoculated in 200 µL of R2A broth (MB cell, Republic of Korea) contained in each well of a 96-well cell culture microplate (SPL Life Sciences, Pocheon-si, Republic of Korea) followed by incubation for 5 days at 28 °C under anaerobic conditions using a GasPak EZ Anaerobe system (BD, USA).

A colorimetric plate screening method modified from the previous studies was used to measure NH_4_^+^ in the culture supernatant [21,22]. Briefly, 10 µL supernatants of the culture in each well were transferred to their corresponding positions on a new 96-well microplate. Consecutively, 90 µL of dH_2_O were added to each well of the plate, generating a 10-fold dilution. Aliquots of 80 µL of NH_4_^+^ reagent A (0.2 M sodium hydroxide, 1 M sodium salicylate, and 5.88 mM sodium nitroprusside dihydrate in dH_2_O), and 20 µL of NH_4_^+^ reagent B (5.1 mM sodium dichloroisocyanurate in dH_2_O) were added to each well of the 96-well microplate. The absorbance at 660 nm was measured using a Multiskan SkyHigh Microplate Spectrophotometer (Thermo Fisher Scientific, Waltham, MA, USA) after 30 min incubation at 25 °C, and the NH_4_^+^ amount was calculated using a series of NH_4_^+^ standard concentrations at 5 mM, 2.5 mM, 1 mM, 0.75 mM, 0.5 mM, 0.25 mM, and 0.1 mM.

### 2.3. Genomic Analyses

The genomic DNA of strain OS1-26 was extracted from the bacterial cell pellets using the DNeasy PowerLyzer Microbial Kit (Qiagen, San Diego, CA, USA) according to the manufacturer’s instructions. The extracted DNA, in 50 µL of elution buffer, was confirmed for purity (1.8~2.0 at 260/280 nm and 2.0~2.2 at 260/230 nm) and concentration (>4000 ng) using a Multiskan SkyHigh Microplate Spectrophotometer with the µDrop Duo Plate (Thermo Fisher Scientific, Waltham, MA, USA).

The 16S rRNA gene was amplified from the genomic DNA by 27F (5′-AGA GTT TGA TCM TGG CTC AG-3′) and 1492R (5′-CGG TTA CCT TGT TAC GAC TT-3′) oligonucleotide primers [23]. The 16S rRNA gene amplicons were sequenced using an ABI 3730XL DNA analyzer (Thermo Fisher Scientific, Waltham, MA, USA), assembled by Solgent. Co. Ltd. (Daejeon, Republic of Korea), and then, submitted to the NCBI GenBank (under accession no. PQ432427). The whole-genome sequencing with the genomic DNA was conducted by long-read sequencing by the Oxford MinION Nanopore platform (Oxford Nanopore Technologies, Oxford, UK) in KNU NGS Center (Kyungbuk National University, Daegu, Republic of Korea). The sequencing libraries were prepared using the Native Barcoding Kit 24 V14 and sequenced on an R10.4.1 flow cell using a MinION Mk1c device, according to the manufacturer’s manual. Base calling was conducted by using Guppy Basecaller v2.24 [24] and reads < 1 kb, and the worst 5% of the read bases were filtered out by Filtlong v0.2.1 (https://github.com/rrwick/Filtlong). The quality-filtered reads were assembled de novo using Flye v2.9.1 [25]. The assembled genome was submitted to the NCBI GenBank (under accession no. NZ_CP133264), and gene prediction and annotation were conducted by the NCBI Prokaryotic Genome Annotation Pipeline (PGAP) (Table S1) [26].

To determine the taxonomic lineage of strain OS1-26, 16S rRNA gene sequence similarities and average nucleotide identity (ANI) values were calculated between strain OS1-26 and the other *Clostridium* species using NCBI BLASTN and OrthoANIu, respectively [27]. The percentage of conserved proteins (POCP) values between the *Clostridium* spp. genomes were calculated as described by Qin et al. [28]. Phylogenetic trees were constructed based on the 16S rRNA sequences using the maximum likelihood method with a bootstrap analysis (n = 1000) using MEGA X software [29].

### 2.4. Morphological and Physiological Analyses

The colony properties were determined by observing the colonies of strain OS1-26 anaerobically grown on R2A agar (MB cell, Republic of Korea) for 5 days at 28 °C. The cellular structure was visualized using a Hitachi-H7650 transmission electron microscope (Hitachi, Tokyo, Japan) by the Center for University-Wide Research Facilities (Jeonbuk National University, Jeonju, Republic of Korea). Gram staining was performed using a Gram Color Kit (MB cell, Republic of Korea). Various temperature (10, 25, 30, 35, and 40 °C), pH (4, 5, 6, 7, 8, 9, and 10), and NaCl (0, 0.5, 1, 1.5, and 2%) conditions were tested for growth in R2A broth contained in Hungate anaerobic culture tubes (Chemglass, Vineland, NJ, USA) flushed and filled with N_2_ during 6 days of incubation at 200 rpm in an orbital shaker. A carbon utilization test was conducted using API 50 CH Kit (bioMérieux, Marcy-l’Étoile, France). The cellular fatty acid composition was analyzed by the Korean Culture Center of Microorganisms (Seoul, Republic of Korea) using the MIDI protocol [30].

### 2.5. Extracellular NH_4_^+^ Accumulation by Bacteria in the Culture Medium

The colonies of strains OS1-26, KCTC 1674^T^, and KCTC 1790^T^ were inoculated into R2A broth contained in a Hungate anaerobic culture tube (Chemglass, Vineland, NJ, USA) flushed and filled with N_2_ and then incubated at 30 °C for 96 h. The optical density at 600 nm wavelength (OD_600_) of the bacterial culture was measured at 0, 12, 24, 48, 72, and 96 h by using the GENESYS 30 Visible Spectrophotometer (Thermo Fisher Scientific, Waltham, MA, USA), followed by a collection of the culture supernatant for NH_4_^+^ measurement, as well as cells of the strain OS1-26 for *nifH* transcript analysis. The concentration of NH_4_^+^ in the culture supernatant was measured using the modified colorimetric plate screening method described above. To confirm that the extracellular accumulation of NH_4_^+^ was due to the BNF of strain OS1-26, the whole-cell reaction experiment was conducted since we were unable to grow the strain in a minimal medium, likely due to the missing components required for its cell division. Cells of the strain OS1-26 collected during the log phase of growth in R2A broth (MB cell, Republic of Korea) were harvested and washed twice with PBS (pH 7.0), and the washed cells were then inoculated into 100 µL of a defined minimal medium containing 0.5 g dextrose, 0.5 g soluble starch, 0.3 g K_2_HPO_4_, 0.05 g MgSO_4_·7H_2_O, 0.3 g sodium pyruvate, 0.01 g FeSO_4_, 0.01 g Na_2_MoO_4_·2H_2_O, 0.5 g KH_2_PO_4_, 0.026 g CaCl_2_·2H_2_O per liter, without any fixed nitrogen nutrients, followed by aerobic and anaerobic incubations in 96-well cell culture microplate (SPL Life Sciences, Pocheon-si, Republic of Korea) at 30 °C for 3, 12, and 24 h. Anaerobic incubation was conducted using a GasPak EZ Anaerobe system (BD, USA). Subsequently, the OD_600_ and NH_4_^+^ concentrations in the culture supernatant were measured. All experiments were conducted in triplicate.

### 2.6. Transcript Analyses

The total RNA was extracted from the cells anaerobically cultured in R2A broth by using a TRIzol Max Bacterial RNA Isolation Kit (Thermo Fisher Scientific, Waltham, MA, USA) in accordance with the manufacturer’s instructions. First-strand cDNA was synthesized from an RNA sample (100 ng) by using the DiaStar RT Kit (SolGent, Daejeon, Republic of Korea) with random hexamers. The oligonucleotide primers lsa_nifH_F (5′-ACT GCG TGG AGT CAG GTG GG-3′) and las_nifH_R (5′-GGA ACT GCA AAA CCG CCG CA-3′) were designed based on the whole-genome sequence of strain OS1-26 and used to amplify transcripts of the *nifH* gene. The reaction mixture for qPCR (10 µL) contained 1X PowerUp SYBR Green Master Mix (Thermo Fisher Scientific, Waltham, MA, USA), 0.2 µM each primer, and 2 µL of cDNA samples. The real-time qPCR was performed using a QuantStudio 1 Real-Time PCR system (Thermo Fisher Scientific, Waltham, MA, USA) with the following conditions: 50 °C for 120 s, and 95 °C for 120 s, followed by 40 cycles of 95 °C for 15 s and 60 °C for 60 s. A melting curve analysis and agarose gel electrophoresis were conducted to confirm the correct amplification of the PCR products. The 16S rRNA was also quantified by qPCR with Eub338 (5′-ACT CCT ACG GGA GGC AGC AG-3′) and Eub518 (5′-ATT ACC GCG G CT GCT GG-3′) primers to normalize the transcript levels of *nifH* gene [31].

### 2.7. Statistical Analyses

The statistical significance between the quantitative data obtained from the extracellular NH_4_^+^ production experiments and the transcript analyses was evaluated using the Wilcoxon test and one-way ANOVA, performed with the PAST software [32].

## 3. Results and Discussion

### 3.1. Genomic Taxonomy

**Figure 1:**
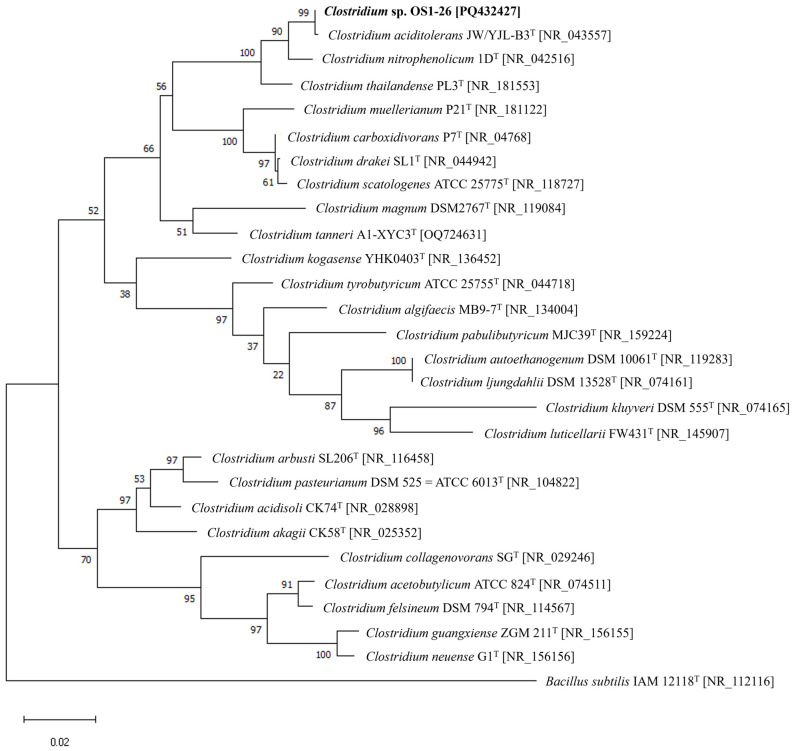
Phylogenetic relationship of *Clostridium* species based on the 16S rRNA gene sequences. The tree was constructed by the maximum-likelihood method using MEGA X software. GenBank accession numbers are shown in square brackets. Bootstrap values (%) were generated from 1000 replicates are shown. Branch lengths correspond to sequence differences as indicated by the scale bar.

The nearly full length (1415 bp) of the 16S rRNA sequence of strain OS1-26 was compared to the others of known-type strains of *Clostridium* species, and a phylogenetic tree with the sequences has been constructed by using the maximum likelihood method (Figure 1). The highest 16S rRNA sequence similarity of strain OS1-26 was observed with *C. aciditolerans* JW/YJL-B3^T^ (99.71%), followed by *C. nitrophenolicum* 1D^T^ (98.52%) and *C. thailandense* PL3^T^ (98.45%). These four reference strains of *Clostridium* spp. were observed to form a clade with high bootstrap values on the phylogenetic tree (Figure 1).

To investigate the overall genomic relationships between the strains, the whole-genome sequence of strain OS1-26 (Table S1) was comparatively analyzed with the others obtained from the NCBI GenBank database. The DNA G + C content of strain OS1-26 was 31.41%, which is similar to the ones of *C. aciditolerans* JW/YJL-B3^T^ (31.28%) and *C. thailandense* PL3^T^ (31.14%). The average nucleotide identity (ANI) values between strain OS1-26 and the type strains of *C. aciditolerans* and *C. thailandense* based on the whole-genome comparisons ranged from 80.35 to 93.07%, with the genome coverage from 28.32 to 54.90% (Table 1), which are below the 95% ANI with 60% genome coverage suggested as a species boundary [33]. Overall, the comparative genomic analysis results indicate that strain OS1-26 should not be classified as *Clostridium aciditolerans,* despite the high 16S rRNA gene sequence similarity.

Interestingly, the percentage of conserved protein (POCP) values between strain OS1-26 and the other *Clostridium* species are below the genus boundary (50%), except for *C. aciditolerans* (Table 1) [28], suggesting that POCP would not be an appropriate standard to define the genus *Clostridium*.

### 3.2. Phenotypic Traits

Strain OS1-26 showed the phenotypic features distinguished from *C. aciditolerans,* despite the close genomic relatedness, as shown in Table 2. For example, while the growth conditions of strain OS1-26 for temperature and pH were 25–35 °C and 5.0–8.0, respectively, *C. aciditolerans* JW/YJL-B3^T^ was reported to grow under a more broad range of temperatures and pH conditions, such as 20–45 °C and 3.8–8.9, respectively. Catalase activity was not observed from strain OS1-26, which was observed from *C. aciditolerans* JW/YJL-B3^T^. Moreover, strain OS1-26 was not able to utilize d-lactose, d-raffinose, glycerol, d-xylose, and d-ribose, which were reported to be used by *C. aciditolerans* JW/YJL-B3^T^, and vice versa for d-trehalose and d-sorbitol. Cellular fatty acid profiles of strain OS1-26 and *C. aciditolerans* JW/YJL-B3^T^ are also distinct from each other (Table S2). While the major fatty acid contents of strain OS1-26 are C_16:0_ and C_14:0_, and the summed feature consisted of C_16:1_ ω7c and C_16:1_ ω6c (35.63%, 25.29%, and 18.84%, respectively), *C. aciditolerans* JW/YJL-B3^T^ had the composition of major fatty acids, such as C_16:0_, C_14:0_, and C_15:0_ (26.1%, 16.1%, and 13.4%, respectively). Taken together, the phenotypic analysis results support that it would not be appropriate to assign strain OS1-26 as a member of *C. aciditolerans*. 

On the other hand, the cell morphology of strain OS1-26 was very similar to *C. aciditolerans* JW/YJL-B3^T^, such as rod-shaped, measuring 0.8–1.0 µm wide and 4.0–10.0 µm long, with ambiguous peritrichous flagellation (Figure S1), which was described for *C. aciditolerans* JW/YJL-B3^T^ previously [35]. Interestingly, the cells of strain OS1-26 were stained Gram negative despite its phylogenetic position indicating a Gram-positive bacterium. Since *C. aciditolerans* JW/YJL-B3^T^ was reported to be stained Gram-negative but determined as Gram-type positive based on the Gram-positive cell wall structure observed by electron microscopy [35], strain OS1-26 is also expected to be a bacterium Gram-stain negative but is Gram-type positive considering its phylogeny and close relatedness to *C. aciditolerans*.

### 3.3. Biological Nitrogen Fixation (BNF)

**Figure 2:**
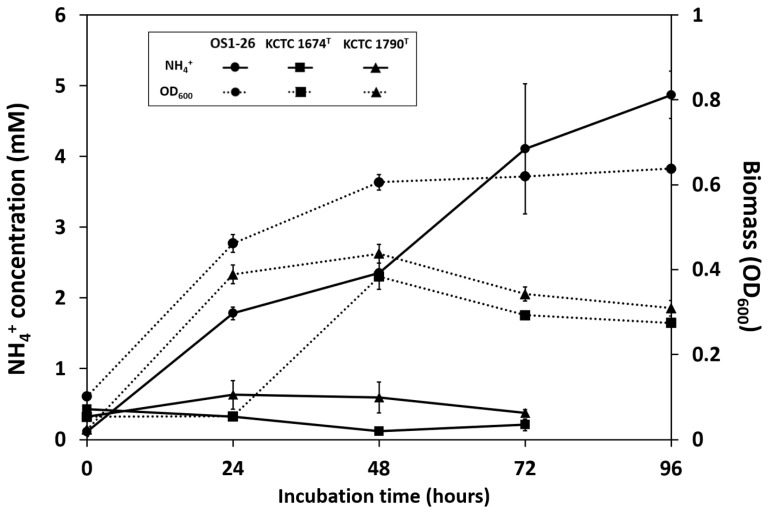
Changes of extracellular NH_4_^+^ concentration and biomass (OD_600_) during the anaerobic cultivation of three *Clostridium* strains, such as OS1-26, KCTC 1674^T^, and KCTC 1790^T^, by incubation time in R2A medium.

The growth and extracellular NH_4_^+^ production of three *Clostridium* diazotrophs, including strain OS1-26 in the R2A broth during anaerobic cultivation, are shown in Figure 2. While significant accumulations of extracellular NH_4_^+^ in the culture supernatant were observed every 24 h for strain OS1-26 (Wilcoxon tests, *p*-values < 0.05), the other well-known diazotrophs in the genus *Clostiridum,* such as *C. pasteurianum* KCTC 1674^T^ and *C. acetobutylicum* KCTC 1790^T^, did not produce extracellular NH_4_^+^ despite of cell growth, as the amounts of extracellular NH_4_^+^ in the culture supernatants were not significantly different between the sampling time points (Wilcoxon tests, *p*-values > 0.05), indicating that the regulation of N fixation by strain OS1-26 should be different from ones of the other two diazotrophs. In general, diazotrophs seem to prioritize using fixed N compounds in order to avoid BNF accompanied by high energy and electron costs [16,40]. It is practical not to produce more NH_4_^+^ than is needed for the diazotrophs themselves. Thus, no accumulation of extracellular NH_4_^+^ in the cultures of strains KCTC 1674^T^ and KCTC 1790^T^ is sensible since the R2A broth is a complex culture medium containing various N nutrients, such as yeast extract and partially hydrolyzed proteins. Due to the inability of strain OS1-26 to grow in the minimal medium, metabolically active cells were collected at the log phase during growth in the R2A medium and transferred into a defined minimal medium without fixed nitrogen nutrients for a whole-cell reaction to assess the biological nitrogen fixation (BNF). Strain OS1-26 continuously accumulated extracellular NH_4_^+^ over time, even without any fixed nitrogen present under anaerobic conditions (Table S3), suggesting that the strain excreted NH4+ produced through BNF. In contrast, inactive cells of the strain OS1-26 under aerobic conditions did not produce any extracellular NH_4_^+^, indicating that the cells were metabolically active despite the strain’s inability to grow in the minimal medium, and extracellular NH_4_^+^ accumulation was not caused by a passive release of stored NH_4_^+^ from the cells under anaerobic conditions.

**Figure 3:**
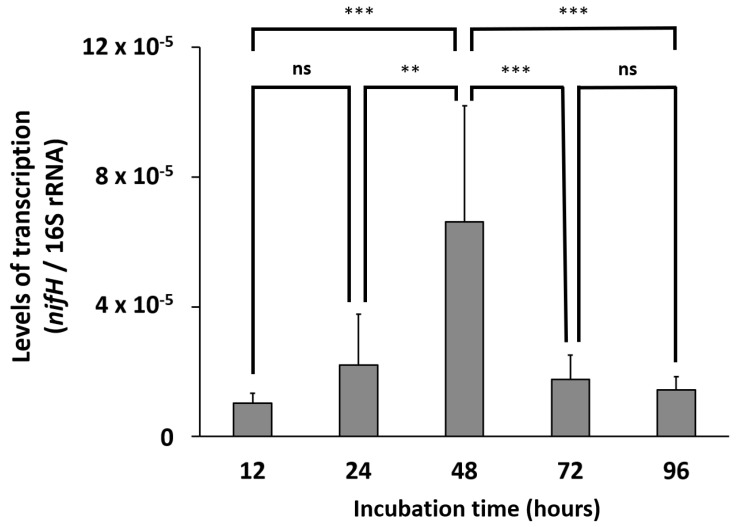
Transcription levels of *nifH* gene of *Clostridium* sp. OS1-26 during the anaerobic cultivation in R2A medium. Transcription levels were normalized by the number of 16S rRNA copies. Statistical significance was determined by one-way ANOVA followed by Tukey’s post hoc test. *p* > 0.05 (ns), *p* < 0.01 (**), *p* < 0.001 (***).

The catalytic functional genes involved in BNF were found in the genome of strain OS1-26 (Table S4), and the *nifH* gene was actively transcribed during the anaerobic cultivation of strain OS1-26 with continuous extracellular NH_4_^+^ production (Figure 2 and Figure 3). As shown in Figure 3, the transcription levels of the *nifH* gene were significantly different between the incubation times (one-way ANOVA, *p*-values < 0.001). The transcription level of the *nifH* gene significantly dropped after 48 h when cell growth ceased at the stationary phase, suggesting that there would be a transcriptional regulation for nitrogenase of strain OS1-26 related to cell growth stages. *Crocosphaera*, a major unicellular diazotroph in the ocean, has been reported to fix N, even when significant amounts of external ammonium are available. BNF, despite the high energy expenditure, was suggested to give *Crocosphaera* a competitive advantage, as it expands their niche [17]. Similarly, the BNF unaffected by other external fixed N may provide strain OS1-26 with an expanded ecological niche in the community and enhance its survival and growth in the soil habitat. This BNF trait of the soil bacterial strain can be further studied for the development of biological N fertilizer using a free-living diazotroph, which can reduce the need for synthetic fertilizers often derived from nonrenewable resources and involve energy-intensive production processes [41].

Regulation of BNF in strain OS1-26 can be predicted since some of the previously reported genes involved in BNF regulation were found in the genome of strain OS1-26. Although the genes encoding AmtB, GlnK, GlnL, GlnK-like protein, and GlnG-like protein were identified (Table S4), the *nifA*, *nifL*, and *glnD* genes were not detected. Since most anaerobic diazotrophs, including *Clostridium*, were reported to lack the *nifA* gene [42], it is unlikely that the regulation of *nif* genes in strain OS1-26 involves the *nifA*/*nifL* system [43]. Additionally, although strain OS1-26 contains the *amtB* gene encoding the bidirectional ammonium transporter [44], which may contribute to extracellular ammonium uptake and excretion, only possible *glnK* (48.83% amino acid sequence similarity to *glnK* of *Clostridium pasteurianum* (GenBank genome locus tag: CPAST_c10620)) was detected in the absence of *glnD*. The genes *glnA*, *glnL* (*ntrB*), and possible *glnG* (*ntrC*) (55.19% amino acid sequence similarity to *glnG* of *Clostridium botulinum* (GenBank genome locus tag: GJ703_03538) were also found. This suggests that the regulation of *nif* genes in OS1-26 is likely controlled by *amtB*-*glnK* and/or *glnA*-*ntrB*-*ntrC* system(s), but differs from the previously reported mechanisms [45,46], based on the sequence dissimilarity of *glnK* and *glnG* with continuous NH_4_^+^ secretion by stain OS1-26. Further studies are required to clarify the BNF regulation in strain OS1-26.

Although BNF by *C. aciditolerans* JW/YJL-B3^T^ has never been described before, its genome was observed to harbor *nifH,* which shows a high nucleotide sequence similarity of 94.68% to one of strain OS1-26 (Table S5), suggesting that a similar BNF trait would be shared between the two *Clostridium* strains with close relatedness in the evolutionary history. On the other hand, the *nifH* gene nucleotide sequences of *C. pasteurianum* and *C. acetobutylicum* are substantially different from one of strain OS1-26 (Table S5), supporting that their BNF likely evolved distantly with different regulations and characteristics.

## 4. Conclusions

Although the 16S rRNA sequence similarity between OS1-26 and *C. aciditolerans* JW/YJL-B3^T^ was above the species boundary cutoff value of 98.65%, the overall genomic relatedness did not satisfy the requirement for species definition, such as >95% ANI with >60% genome coverage. Moreover, strain OS1-26 showed the cellular fatty acid composition and phenotypic characteristics to be distinctly different from the other *Clostridium* species, including *C. aciditolerans*. In taxonomic experiments, it is advisable to include control strains to correct for potential errors in the phenotypic data caused by variations in the experimental design. Thus, strain OS1-26 is currently undetermined for species identity within the genus *Clostridium*, suggesting that additional comparative experiments with the closely related control strains must be conducted to identify species of strain OS1-26 in the future. Additionally, the BNF trait of strain OS1-26 distinguished from the ones of the well-known diazotrophic *Clostridium* species, such as *C. pasteurianum and C. acetobutylicum* has great potential as a biological N fertilizer. As properties of the apple orchard soil where strain OS1-26 was isolated showed optimal pH and low EC rate for growth and nitrogenase activity, respectively, the strain would actively contribute to the microbial community as a key N provider. While the amount of NH_4_^+^ produced by strain OS1-26 in this study may be insufficient to support its immediate application as a biofertilizer in agricultural systems, the extracellular ammonium production—independent of other fixed nitrogen compounds in the environment—demonstrates significant potential for further development in future studies. The continuous NH_4_^+^ excretion from strain OS1-26 in the soil would enhance crop productivity and yield by providing a readily available N source and reducing the N limitation to the crops via a synchronized NH_4_^+^ release with plant demand. Moreover, *Clostridium* sp. OS1-26 is a valuable microbial resource worthy of further study for its ecological roles in the soil ecosystem since its unique BNF trait can be pivotal in the terrestrial soil N cycle.

## Figures and Tables

**Table 1:** Average nucleotide identity (ANI) and percentage of conserved proteins (POCP) values between strain OS1-26 and type strains of other *Clostridium* species.

Compared Genomes		ANI (%)	Genome A Coverage for ANI (%)	Genome B Coverage for ANI (%)	POCP (%)
Genome A	Genome B				
*Clostridium* sp. OS1-26 [NZ_CP133264]	*C. aciditolerans* DSM 17425^T^ [GCA_016316925]	93.07	48.68	54.90	69.11
	*C. thailandense* PL3^T^ [GCA_019207025]	80.35	30.59	28.32	24.18
	*C. muellerianum* P21^T^ [GCA_012926525]	76.76	24.45	25.61	14.89
	*C. carboxidivorans* P7^T^[GCA_001038625]	76.88	25.89	26.46	15.26
	*C. drakei* SL1^T^[GCA_003096175]	76.67	24.63	25.43	14.84
	*C. scatologenes* ATCC 25775^T^ [GCA_000968375]	76.73	25.74	26.32	15.19

**Table 2:** Morphological, physiological, and biochemical characteristics of *Clostridium* sp. OS1-26 and its closely related *Clostridium* species strains: 1, *Clostridium* sp. OS1-26 (this study); 2, *Clostridium aciditolerans* JW/YJL-B3^T^ [34,35]; 3, *Clostridium nitrophenolicum* 1D^T^ [36]; 4, *Clostridium thailandense* PL3^T^ [34]; 5, *Clostridium muellerianum* P21^T^ [37]; 6, *Clostridium carboxidibovans* P7^T^ [35,38,39]. -, negative; +, positive; NR, not reported.

Characteristics	1	2	3	4	5	6
Source	Apple orchard soil	Constructed wetland sediment	subsurface soil sample	Peatland sediment	Old hay	Agricultural settling lagoon
Gram stain	-	-	+	+	+	+
Cell size (μm)	0.8–1.0 × 4.0–10.0	0.5–1.0 × 3.0–9.0	0.6–0.9 × 3.5–5.0	0.8–1.0 × 4.0–10.0	0.4 × 5.5–9.2	0.5 × 3.0
Growth temperature (optimum; °C)	25–35 (30–35)	20–45 (35)	20–45 (30)	20–40 (34)	20–45 (30–40)	24–42 (37–40)
pH (optimum)	5.0–8.0 (6.0–8.0)	3.8–8.9 (7.5)	6.5–8.0 (7.2)	6.0–7.5 (7.0)	5.0–8.5 (6.5)	4.4–7.6 (5.0–7.0)
Growth NaCl (optimum; %)	0–0.5	0–1.5 (0)	0–1	0–1.5 (0.5)	0–20 (0)	NR
Catalase activity	-	+	-	-	-	-
Substrate utilization:						
d-Lactose	-	+	-	+	+	-
Maltose	+	+	-	+	-	-
d-Raffinose	-	+	-	+	NR	-
d-Trehalose	+	-	NR	-	-	+
l-Rhamnose	-	-	+	+	-	NR
d-Sucrose	+	+	-	-	NR	+
d-Sorbitol	weak	-	NR	+	-	-
l-Arabinose	-	-	NR	+	NR	NR
Glycerol	-	+	NR	+	-	+
d-Xylose	-	+	-	+	NR	+
d-Ribose	-	+	-	+	NR	+
d-Glucose	+	+	-	NR	NR	+
Cellobiose	weak	NR	-	NR	NR	+
d-Fructose	+	NR	NR	NR	+	+
d-Mannose	+	+	-	NR	NR	+
Esculin	+	NR	NR	NR	NR	NR

## Data Availability

The original contributions presented in this study are included in the article/supplementary material. Further inquiries can be directed to the corresponding author.

## Supplementary Materials

The following supporting information can be downloaded at https://www.mdpi.com/article/10.3390/microorganisms12122634/s1: Table S1: Genome sequencing summary for *Clostridium* sp. OS1-26; Table S2: Cellular fatty acid contents (% of total fatty acids) of *Clostridium* species type strains: 1, *Clostridium* sp. OS1-26 (this study); 2, *Clostridium aciditolerans* JW/YJL-B3^T^ [34]; 3, *Clostridium nitrophenolicum* 1D^T^ [36]; 4, *Clostridium thailandense* PL3^T^ [34]; 5, *Clostridium muellerianum* P21^T^ [37]; 5, *Clostridium carboxidibovans* P7^T^ [38,39]. -, Negative; +, positive; NR, not reported; Table S3: Changes of extracellular NH_4_^+^ concentration and Biomass (OD_600_) after anaerobic incubation of *Clostridium* sp. OS1-26 in the defined minimal medium without any fixed N nutrients at 30 °C for 0, 3, 12, 24 h; Table S4: Genes involved in nitrogen fixation found from the genome of *Clostridium* sp. OS1-26 (GenBank accession no. NZ_CP133264); Table S5: Nucleotide sequence similarities of *nifH* gene between strain OS1-26 and the other *Clostridium* species type strains. Locus tags for the *nifH* gene in the Genbank genomes were written in the square brackets behind strain IDs; Figure S1: Transmission electron microscopic image of a cell of *Clostridium* sp. OS1-26.
