# Supplementary material for: Excessive Extracellular Ammonium Production by a Free-Living Nitrogen-Fixing Soil Clostridium sp. Strain
Source: Microorganisms. 2024 Dec 19;12(12):2634. doi: 10.3390/microorganisms12122634 (PMC11679708; doi:10.3390/microorganisms12122634)
Supplement: Supplementary file 1 [file microorganisms-12-02634-s001.zip › microorganisms-3322582-supplementary.pdf]

Supplementary Information

Excessive Extracellular Ammonium Production by a Free-Living Nitrogen-Fixing Soil *Clostridium* sp. Strain

Soyeon Park and Jeonghwan Jang *

Division of Biotechnology and Advanced Institute of Environment and Bioscience, Jeonbuk National University, Iksan 54596, Jeonbuk, Republic of Korea; psu1143@naver.com

* Correspondence: jangj@jbnu.ac.kr

Table S1. Genome sequencing summary for *Clostridium* sp. OS1-26.

Attribute	Value
Total sequence reads	5,878,738 bp
Number of contigs	1
Total length of contigs	5,878,738 bp
Max length of contig	5,878,738 bp
Genome coverage	247×
Number of CDSs	5,652
Number of tRNAs	104
Number of rRNA genes (5S, 16S, 23S)	13, 15, 14
GenBank accession no.	NZ_CP133264

Table S2. Cellular fatty acid contents (% of total fatty acids) of *Clostridium* species type strains: 1, *Clostridium* sp. OS1-26 (this study); 2, *Clostridium aciditolerans* JW/YJL-B3^T (Chaikitkaew*, et al.*, 2022); 3, *Clostridium nitrophenolicum* 1D^T (Suresh*, et al.*, 2007); 4, *Clostridium thailandense* PL3^T (Chaikitkaew, et al., 2022); 5, *Clostridium muellerianum* P21^T (Doyle*, et al.*, 2022); 5, *Clostridium carboxidibovans* P7^T (Liou*, et al.*, 2005, Miller*, et al.*, 2024). -, Negative; +, positive; NR, not reported.

**Cellular fatty acids**	**1**	**2**	**3**	**4**	**5**
$C_{12:0}$	2.02	NR	NR	NR	NR
$C_{13:0}$ iso	0.26	NR	-	NR	NR
$C_{13:1}$ at 12-13	0.44	NR	NR	NR	NR
$C_{13:0}$	0.38	0.9	-	-	NR
$C_{14:0}$ iso	0.57	NR	2.46	NR	NR
$C_{14:0}$	**25.29**	**16.1**	**10.2**	4.2	3.5
$C_{15:0}$ iso	0.29	NR	-	NR	NR
$C_{15:0}$ anteiso	1.02	NR	-	NR	NR
$C_{15:1}$ ω8c	2.39	NR	-	NR	4.4
$C_{15:1}$ ω6c	3.69	NR	-	NR	NR
$C_{15:0}$	---	**13.4**	NR	-	NR
$C_{16:1}$ iso H	0.53	NR	NR	NR	NR
$C_{16:0}$ N alcohol	0.38	NR	NR	NR	NR
$C_{16:0}$ iso	0.29	NR	1.92	NR	NR
$C_{16:1}$ ω5c	1.41	NR	NR	NR	NR
$C_{16:0}$	**35.63**	**26.1**	**28.02**	**33.8**	**41.5**
$C_{17:1}$ ω8c	0.59	NR	-	NR	NR
$C_{17:1}$ ω6c	0.81	NR	4.24	NR	NR
$C_{17:0}$ cyclo	1.42	-	**20.2**	**20.2**	NR
$C_{17:0}$	0.53	NR	-	-	6.0
$C_{18:0}$	0.81	0.9	5.18	0.4	NR
$C_{19:0}$ cyclo ω8c	0.75	NR	NR	NR	NR
Summed feature 1*	0.50	NR	NR	NR	NR
Summed feature 3*	**18.84**	NR	NR	NR	NR
Summed feature 8*	0.56	NR	NR	NR	NR

*Summed feature 1 contained iso-$C_{15:1}$ H and $C_{13:0}$ 3OH; Summed feature 3 contained $C_{16:1}$ ω6c/$C_{16:1}$ ω7c; Summed feature 8 contained $C_{18:1}$ ω6c.

Table S3. Changes of extracellular NH_4^+ concentration and Biomass (OD_{600}) after anaerobic incubation of *Clostridium* sp. OS1-26 in the defined minimal medium without any fixed N nutrients at 30 °C for 0, 3, 12, and 24 hours.

Incubation time (hours)	aerobic incubation		anaerobic incubation	
	OD_{600}	NH_4^+ (mM)	OD_{600}	NH_4^+ (mM)
0	6.23 ± 0.51	0	6.23 ± 0.51	0
3	5.98 ± 0.68	0	6.27 ± 0.75	1.03 ± 0.17
12	6.01 ± 0.21	0	6.17 ± 0.23	4.02 ± 0.25
24	5.81 ± 0.33	0	6.21 ± 0.31	7.56 ± 0.59

Table S4. Genes involved in nitrogen fixation found from the genome of *Clostridium* sp. OS1-26 (GenBank accession no. NZ_CP133264).

Pathway	locus_tag	Gene	Related product
Nitrogen fixation	RCG18_05100	*fixB*	electron transfer fla voprotein subunit al pha/FixB, beta/FixA family protein
	RCG18_05105	*fixA*	
	RCG18_11280	*fixA*	
	RCG18_11285	*fixB*	
	RCG18_22260	*fixA*	
	RCG18_22425	*fixA*	
	RCG18_24820	*fixB*	
	RCG18_24825	*fixA*	
	RCG18_25475	*fixB*	
	RCG18_25480	*fixA*	
	RCG18_22535	*fixH*	FixH family protein
	RCG18_11940	*nifS*	cysteine desulfurase
	RCG18_23145	*nifS*	
	RCG18_24770	*sufS*	
	RCG18_28830	*nifS*	
	RCG18_10115	*nifB*	NifB/NifX family molybdenum-iron cluster-binding protein
	RCG18_14355	*nifB*	
	RCG18_01850	*nifJ*	pyruvate:ferredoxin (flavodoxin) oxidoreductase
	RCG18_04330	*nifJ*	
	RCG18_14340	*nifD*	nitrogenase reductase
	RCG18_14345	*nifK*	
	RCG18_14350	*nifH*	
	RCG18_28825	*nifU*	Fe-S cluster assemb ly protein
	RCG18_24765	*sufU*	
	RCG18_24775	*sufD*	
	RCG18_24780	*sufB*	
	RCG18_24785	*sufC*	Fe-S cluster assembly ATPase SufC
Regulation of nitrogen metabolism	RCG18_04885	*amtB*	ammonium transporter
	RCG18_05325 + RCG18_05330	*glnK* (possibly)	sensor histidine kinase
	RCG18_05335	*glnL* or *ntrB*	response regulator
	RCG18_06600	*glnA*	glutamine synthetase
	RCG18_07820 + RCG18_07825	*glnG* or *ntrC* (possibly)	sigma-54 dependent transcriptional regulator

Table S5. Nucleotide sequence similarities of *nifH* gene between strain OS1-26 and the other *Clostridium* species type strains. Locus tags for the *nifH* gene in the Genbank genomes were written in the square brackets behind strain IDs.

	Nucelotide sequence similarity % (query coverage %)		
	Clostridium aciditolerans DSM17425^T [I6U51_RS03855]	*Clostridium pasteurianum* DSM525^T [AQ983_RS05415]	*Clostridium acetobutylicum* ATCC824^T [CA_RS01415]
Clostridium sp. OS1-26 [RCG18_RS14350]	94.68% (98%)	67.56% (49%)	66.33% (49%)

Figure S1. Transmission electron microscopic image of a cell of *Clostridium* sp. OS1-26.

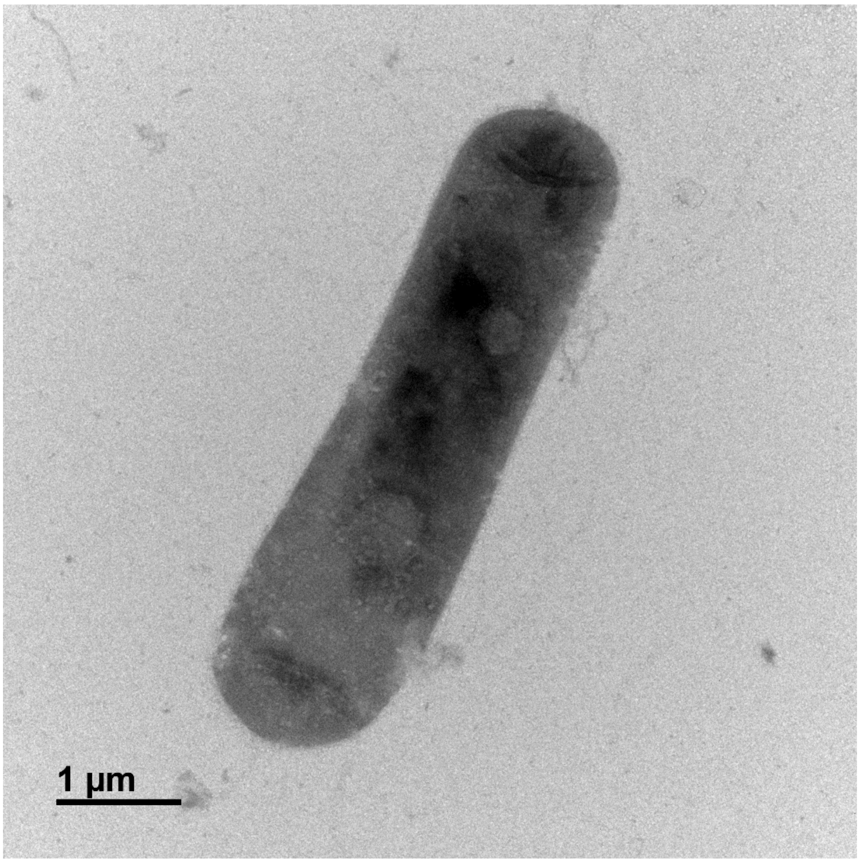

References

Chaikitkaew, S., In-Chan, S., Singkhala, A., Tukanghan, W., Mamimin, C., Reungsang, A., Birkeland, N.-K., & O-Thong, S. (2022). *Clostridium thailandense* sp. nov., a novel CO_2-reducing acetogenic bacterium isolated from peatland soil. *International Journal of Systematic and Evolutionary Microbiology,* 72, 005254.

Doyle, D. A., Smith, P. R., Lawson, P. A., & Tanner, R. S. (2022). *Clostridium muellerianum* sp. nov., a carbon monoxide-oxidizing acetogen isolated from old hay. *International Journal of Systematic and Evolutionary Microbiology,* 72, 005297.

Liou, J. S.-C., Balkwill, D. L., Drake, G. R., & Tanner, R. S. (2005). *Clostridium carboxidivorans* sp. nov., a solvent-producing clostridium isolated from an agricultural settling lagoon, and reclassification of the acetogen *clostridium scatologenes* strain SL1 as *clostridium drakei* sp. nov. *International journal of systematic and evolutionary microbiology,* 55, 2085-2091.

Miller, S., Hendry, M., King, J., Sankaranarayanan, K., & Lawson, P. A. (2024). *Clostridium tanneri* sp. nov., isolated from the faecal material of an alpaca. *International Journal of Systematic and Evolutionary Microbiology,* 74, 006372.

Suresh, K., Prakash, D., Rastogi, N., & Jain, R. (2007). *Clostridium nitrophenolicum* sp. nov., a novel anaerobic *p*-nitrophenol-degrading bacterium, isolated from a subsurface soil sample. *International Journal of Systematic and Evolutionary Microbiology,* 57, 1886-1890.
